# Development of a hybrid 2.5D deep learning model for glioma survival prediction using T1-weighted MRI from the CGGA database

**DOI:** 10.1016/j.ejro.2025.100697

**Published:** 2025-10-26

**Authors:** Kai Jin, Caixing Sun, Liang Xia

**Affiliations:** Department of Neurosurgery, Zhejiang Cancer Hospital, Hangzhou Institute of Medicine (HIM), Chinese Academy of Sciences, Hangzhou, Zhejiang 310022, China

**Keywords:** 2.5D deep learning (2.5D_DL), MRI, CGGA, Glioma, Survival prediction

## Abstract

**Background:**

Current glioma survival prediction relies on invasive molecular profiling. To overcome this, a non-invasive deep learning framework using T1-weighted contrast-enhanced MRI (T1CE) was developed to predict overall survival. This framework addresses computational limitations associated with the volumetric analysis while preserving important spatial information.

**Methods:**

We designed a hybrid 2.5D convolutional neural network to process multi-slice inputs, including the center slice and its adjacent slices, from 217 patients in the CGGA database. Transfer learning using ResNet and DenseNet architectures were employed to initialize the models. These models were subsequently fine-tuned with the Cox proportional hazards loss function. After the fine-tuning process was completed, the imaging signature was combined with clinical and molecular variables, including IDH and 1p19q status, to build an integrated model. Performance was evaluated via C-index, time-dependent AUC, and Kaplan-Meier analysis in independent training (70 %) and testing (30 %) cohorts.

**Results:**

The Combined model achieved superior discrimination, with a training C-index of 0.819 (95 % CI: 0.758–0.880) and a testing C-index of 0.804 (95 % CI: 0.708–0.900). It significantly outperformed the isolated Radiomic, deep learning (2D and 2.5D), and Clinical models (all p < 0.05). Moreover, time-dependent ROC analysis demonstrated consistent model performance over 1–5 years, with AUC values ranging from 0.851 to 0.906. The stratified survival curves clearly revealed distinct prognostic groups (log-rank p < 0.001).

**Conclusions:**

The 2.5D multi-source framework provides a clinically feasible, non-invasive tool for preoperative survival prediction, enabling personalized therapeutic strategies for glioma patients.

## 1 Introduction

Gliomas are the most common type of primary brain tumors. They exhibit considerable variability in their biological behavior and clinical outcomes [1]. This variability highlights the need for effective prognostic tools that can help tailor treatment strategies for individual patients. Currently, prognosis is largely based on the WHO classification, which combines histopathological evaluations with molecular profiling. This molecular profiling includes the assessment of IDH mutation status and 1p/19q codeletion to categorize risk levels. However, acquiring these biomarkers typically requires invasive procedures like surgical resection or biopsy. These procedures carry risks including infection and neurological damage, and may not be suitable for all patients [2]. As a result, there is a growing focus on developing non-invasive prognostic tools derived from magnetic resonance imaging (MRI), which is commonly used in clinical settings.

Multiparametric MRI techniques include T1-weighted contrast-enhanced imaging, T2-weighted imaging, FLAIR, diffusion, and perfusion sequences. These methods enable detailed in vivo analysis of glioma characteristics by revealing tumor structure, blood-brain barrier integrity, cellular density, and blood flow dynamics [3], [4]. Among these, T1-weighted contrast-enhanced imaging is particularly useful for identifying disruptions in the blood-brain barrier, which are signs often associated with more aggressive tumor types. Volumetric MRI data provide rich information but pose significant computational challenges when used for deep learning-based survival predictions. consequently, the computational demand to process full three-dimensional datasets often exceeds the capabilities of available hardware. As a result, researchers either work with individual two-dimensional slices, which can lose important spatial information, or use network architectures that are less effective at capturing three-dimensional spatial features [5].

Traditional radiomics and machine learning methods have shown some initial promise in predicting outcomes for glioma patients. However, their application in clinical settings faces challenges due to inconsistencies in methodology, varying imaging protocols, and limited abilities to generalize across different populations. Neural network-based survival models, such as DeepSurv and NMTLR, show potential by effectively capturing complex, non-linear relationships in high-dimensional data and by offering advantages over standard Cox regression models. Although deep learning has advanced survival prediction in other cancer imaging fields, its application in glioma prognosis—particularly with advanced multiparametric MRI—remains underexplored, primarily because 3D data demands high computational cost and hardware resources [6], [7], [8]. To address this issue, we propose a novel 2.5D deep learning framework that leverages multiparametric MRI, with particular emphasis on T1ce sequences, to predict overall survival in glioma patients [9].

By integrating slice information from multiple adjacent slices, 2.5D deep learning models represent a significant evolution from their 2D predecessors. This approach benefits medical imaging by providing a more comprehensive understanding of anatomical structures. The 2.5D model effectively bridges the gap between the simplicity of 2D CNNs and the high computational expense of full 3D models. It offers a balanced solution that leverages depth information without requiring excessive computational resources. A prime example of 2.5D CNN model is its application in segmenting contrast-enhanced lesions in brain MRI scans [10], [11]. Here, "2.5D" refers to a method that processes individual 2D slices but incorporates contextual information from adjacent slices to capture some 3D spatial information. This method balances computational efficiency with the preservation of critical spatial context within the imaging data. Our framework aims to create a reliable, non-invasive prognostic tool that can improve clinical decision-making.

## 2 Methods

### 2.1 Patients

**Fig. 1:**
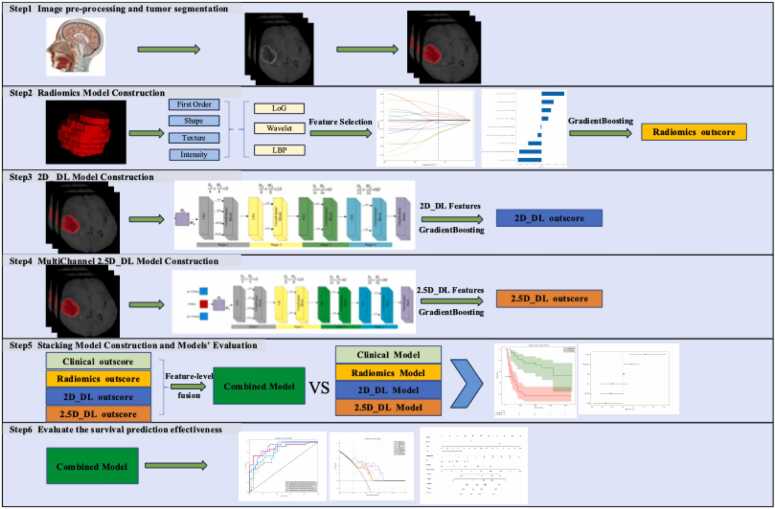
Workflow of this study.

**Fig. 2:**
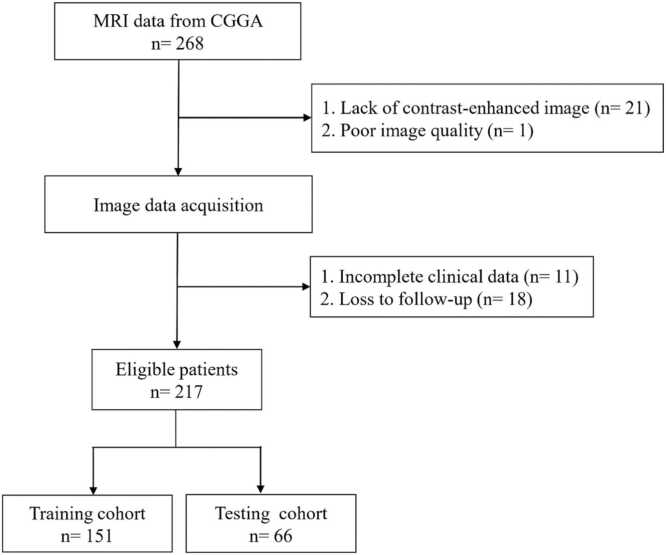
Flowchart of the criteria for patient selection.

The research flowchart is illustrated in Fig. 1. The dataset used in this work is collected from CGGA (Chinese Glioma Genome Atlas) [12], which is a single-center dataset. The collected data were normalized, and samples with defective clinical and follow-up information were eliminated. The images with poor quality or lack of contrast-enhanced series were also excluded. There were 217 samples left after filtration. This study divided the samples into two cohorts randomly: a training cohort, comprising 70 % of the data, and a testing cohort, consisting of the remaining 30 % (Fig. 2). Results of the two cohort comparisons (p > 0.05) indicated that there were no significant differences between the two groups, that we will conduct further analysis after increasing the sample size in the future (Table 1).

**Table 1:** Baseline characters of our cohorts.

Feature	ALL	test	train	P-value
Age	43.89 ± 12.26	43.39 ± 11.63	44.11 ± 12.56	0.754
Histology				0.635
Non-GBM	82(37.79)	27(40.91)	55(36.42)	
GBM	135(62.21)	39(59.09)	96(63.58)	
Grade				0.76
WHO II	76(35.02)	23(34.85)	53(35.10)	
WHO III	59(27.19)	16(24.24)	43(28.48)	
WHO IV	82(37.79)	27(40.91)	55(36.42)	
Gender				0.295
Female	82(37.79)	21(31.82)	61(40.40)	
Male	135(62.21)	45(68.18)	90(59.60)	
Radiotherapy				0.872
No	30(13.82)	10(15.15)	20(13.25)	
Yes	187(86.18)	56(84.85)	131(86.75)	
Chemotherapy				0.652
No	79(36.41)	26(39.39)	53(35.10)	
Yes	138(63.59)	40(60.61)	98(64.90)	
IDH				0.969
Wildtype	104(47.93)	31(46.97)	73(48.34)	
Mutant	113(52.07)	35(53.03)	78(51.66)	
1p19q codeletion				0.249
No	185(85.25)	53(80.30)	132(87.42)	
Yes	32(14.75)	13(19.70)	19(12.58)	
MGMTp methylation				0.652
No	138(63.59)	40(60.61)	98(64.90)	
Yes	79(36.41)	26(39.39)	53(35.10)	

### 2.2 Image acquisition

MRI scans for these patients were performed using 3.0-T Trio scanners (Siemens). The protocol typically included axial T1-weighted scans with repetition times of 156–2520 ms, echo times of 2–19.7 ms, and slice thicknesses of 3–5 mm. Additionally, contrast-enhanced (CE) scans were acquired following the administration of 0.1 mmol/kg of Gd-DTPA contrast agent (Beijing Beilu Pharmaceutical Co., Beijing, China), with repetition times of 560–2520 ms, echo times of 2.3–19.7 ms, and slice thicknesses of 3–5 mm.

ROI Segmentation: In our study, two experienced radiologists used ITK-SNAP to manually delineate the ROI on the training dataset images. To ensure accuracy, a senior radiologist with over 15 years of experience reviewed the annotations made by both radiologists and addressed any discrepancies that arose. This meticulous manual delineation process served as the foundation for the training data used to develop our automated segmentation model.

### 2.3 Radiomics procedure

#### 2.3.1 Feature extraction

Handcrafted features were divided into three main categories: Geometry, related to tumor shape; Intensity, concerning voxel intensity distribution; and Texture, which analyzes advanced intensity patterns, including those from the Gray Level Co-occurrence Matrix (GLCM). The process of feature extraction adhered to the guidelines set forth by the Imaging Biomarker Standardization Initiative (IBSI) and utilized the PyRadiomics tool, specifically version 3.0.1. To maintain consistency in texture feature analysis, images were resampled using cubic interpolation to achieve isotropic voxel spacing of 1 mm × 1 mm × 1 mm. Before feature extraction, intensity values were discretized into 64 gray levels. For multi-center standardization studies, the Standardized Environment for Radiomics Analysis (SERA), a MATLAB-based framework compliant with the IBSI standards was employed to ensure consistency across datasets. To focus the analysis on relevant tumor characteristics, ROIs were manually delineated. From each ROI, a total of 225 radiomic features were extracted.

### 2.4 Feature selection

Intraclass Correlation Coefficient (ICC): We evaluated the consistency of feature extraction amid segmentation uncertainties through test-retest and inter-rater reliability analyses. Specifically, one rater performed two segmentations on the same randomly selected sample of 30 patients to assess test-retest reliability. For inter-rater reliability, two independent raters segmented the subregions within the Volumes of Interest (VOIs) in a separate group of 30 patients. The ICC was employed to assess the reliability of radiomic features derived from these segmented subregions. Features with ICC ≥ 0.85 were considered reliable despite segmentation variability.

Correlation Analysis: Pearson's correlation coefficient was used to analyze features with high test-retest reliability. If two features had a correlation coefficient greater than 0.8, one was retained based on its clinical relevance as determined by expert knowledge, to avoid redundancy. Then, a recursive feature elimination strategy removed the feature with the highest average correlation to others in each iteration.

Univariate Cox Regression: To streamline the extensive feature set, we implemented univariate Cox regression analysis. We ranked features by their p-values and selected a number of top features equal to the size of the training sample. This choice helps to balance model complexity and to avoid overfitting. Our experiments showed that this feature selection method outperformed using all features whose p-values were below 0.05.

Lasso-Cox Regression: The final feature set for the radiomic signature was determined using LASSO (Least Absolute Shrinkage and Selection Operator) Cox regression. This technique identifies irrelevant features by zeroing their coefficients, which depends on the regularization parameter λ. The optimal regularization parameter λ was determined by 10-fold cross-validation, selecting the value that minimized the cross-validated partial likelihood deviance.

### 2.5 Deep learning procedure

#### 2.5.1 Data preparation

Data Preprocessing: This study addressed challenges in medical image analysis by standardizing voxel spacing in Volumes of Interest (VOIs) to 1 × 1 × 1 mm using a linear interpolation resampling method. This standardization improved the consistency of image quality and the accuracy of feature extraction in the analysis.

#### 2.5.2 Model training

Image Preparation: To effectively leverage 2.5D data (e.g., volumetric medical images with limited slices), we adopted a multi-view ROI extraction strategy. For each 3D volume, ROIs were extracted ±1 from the central slice and include the central slice. These 2D slices were then stacked along the channel dimension to form a multi-channel input, preserving spatial context and enabling compatibility with standard 2D convolutional architectures. This approach combines cross-sectional features while reducing the memory requirements associated with full 3D processing.

Transfer learning: We evaluated five well-established architectures—ResNet50, ResNet101, Inception-V3, DenseNet201, and DenseNet161—initialized with weights pretrained on ImageNet. Transfer learning was employed to adapt these models to our domain-specific task, with the following modifications:

Input Layer Adjustment: The first convolutional layer was reconfigured to accept multi-channel inputs (e.g., 3 channels for 1 planes × 3 slices per ROI).
Feature Fusion: Late-stage features from all architectures were fed into a fully connected layer with L2 regularization, followed by a single-node output for log-risk prediction.

We conducted a comparative analysis to identify the optimal backbone neural network by evaluating training stability, convergence speed, and discriminative performance. The discriminative performance was assessed using the concordance index (C-index).

Objective Function: Cox Proportional Hazards Loss: The model was trained to minimize the negative partial log-likelihood (Cox loss), which quantifies the alignment between predicted risks and observed survival times:

(1) $$ℒ\left(\theta \right)=-\frac{1}{N_{E}}\sum_{i:\delta_{i}=1}\left(h_{\theta }\left(x_{i}\right)-\;\log\sum_{j\in R\left(T_{i}\right)}eh\theta (xj)\right)$$

Training Protocol:

Warm-up: Early layers were frozen for 5 epochs to stabilize feature extraction.
Fine-tuning: All layers were unfrozen, with gradient clipping (norm=1.0) to prevent explosion.
Early Stopping: Monitored validation loss (patience=10 epochs).

#### 2.5.3 Signature building

Deep learning signature (DL Signature): is derived from a neural network trained using the partial likelihood loss function of the Cox proportional hazards model to capture non-linear relationships between imaging-derived features and survival outcomes. The training hyperparameters of 2D and 2.5D deep learning were set as a batch size of 32, epoch of 40, and stochastic gradient descent (SGD) optimizer with a learning rate of 0.01.

#### 2.5.4 Metrics

Our research employed sophisticated statistical and computational techniques to address prevailing challenges in medical image analysis. Specifically, we constructed Cox proportional hazards models with L2 regularization for survival analysis. We then performed Kaplan-Meier analysis on samples stratified into high-risk and low-risk groups according to their predicted hazard ratios (HRs). The significance of group separation was assessed via a multivariate log-rank test.

### 2.6 Construction of the fusion model

The current study employed feature-level fusion strategies to establish the fusion model. Feature-level fusion, also known as early fusion, involves concatenating features from different data modalities into a single feature vector. The radiomics features of the primary tumor were extracted using PyRadiomics, while the 2D/2.5D_DL features were obtained through deep convolutional neural networks (DCNNs), as described in the Methods section. These features, along with clinical and radiological characteristics, were standardized using z-score normalization. Subsequently, Spearman correlation analysis, ICC, and LASSO regression were performed to select the most relevant features. Finally, the support vector machine (SVM) classifier was trained to construct the feature-level fusion model, known as the combined model.

### 2.7 Statistical analysis

The normality of clinical features was assessed using the Shapiro-Wilk test. Continuous variables were subjected to the independent samples *t*-test or the Mann-Whitney *U* test based on their distribution characteristics. Categorical variables were examined using Chi-square (χ²) tests. The baseline characteristics across all cohorts are detailed in Table 1. Notably, p-values exceeding 0.05 between cohorts indicated no significant differences, which suggests that the division between cohorts was unbiased.

All data analyses were performed on the OnekeyAI platform version 4.9.1 utilizing Python 3.7.12. Statistical assessments were conducted with Statsmodels version 0.13.2, and radiomics features were extracted using PyRadiomics version 3.0.1. Machine learning models, such as SVM were implemented with Scikit-learn version 1.0.2. Our deep learning frameworks were developed using PyTorch version 1.11.0, with performance improvements achieved through optimization leveraging CUDA version 11.3.1 and cuDNN version 8.2.1.

## 3 Results

### 3.1 Clinical signature

In our study, we initially evaluated clinical features, from which multivariate analysis identified 3 significant characteristics (Table 2). These features were instrumental in constructing the clinical model.

**Table 2:** Univariable and multivariable analysis of clinical features.

Feature	Univariate analysis			Multivariate analysis		
	HR	95 %CI	p-value	HR	95 %CI	P-value
Histology	0.286	0.19–0.429	< 0.05	0.705	0.488–1.081	0.062
Grade	2.375	1.84–3.065	< 0.05	1.368	1.103–1.697	< 0.05
Gender	0.764	0.512–1.141	0.189			
Age	1.048	1.031–1.066	< 0.05	1.021	1.008–1.034	< 0.05
Radiotherapy	1.522	0.783–2.958	0.215			
Chemotherapy	1.18	0.772–1.804	0.444			
IDH mutation	0.299	0.197–0.453	< 0.05	0.678	0.482–0.952	< 0.05
1p19q codeletion	0.259	0.114–0.588	< 0.05	0.635	0.371–1.087	0.098
MGMTp methylation	0.869	0.572–1.32	0.510			

### 3.2 Survival analysis

**Fig. 3:**
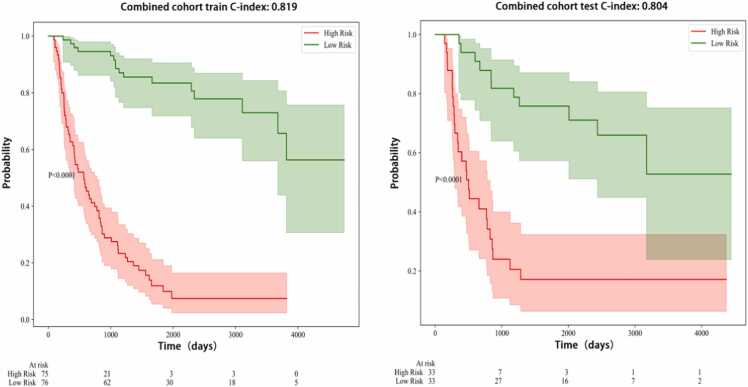
KM of Combined Signature.

Among the five models, DenseNet-161 showed the best prediction performance based on its output scores. The Area Under the Curve (AUC) values of different indicators were calculated separately in the training and test cohorts to assess their predictive performance. In the training cohort, the Combined indicators-which integrate clinical, radiomics, and deep learning features—demonstrated the highest AUC at 0.819 (95 % CI: 0.758–0.880), followed by the Clinical indicator at 0.797 (95 % CI: 0.733–0.861). Radiomics indicators showed an AUC of 0.764 (95 % CI: 0.696–0.832), while the 2D_DL and 2.5D_DL indicators showed AUCs of 0.685 (95 % CI: 0.611–0.759) and 0.739 (95 % CI: 0.669–0.809), respectively. Meanwhile, in the test cohort, the Combined indicators maintained the highest AUC at 0.804 (95 % CI: 0.708–0.900), with Clinical indicators closely following at 0.799 (95 % CI: 0.703–0.896). Moreover, Radiomics indicators exhibited an AUC of 0.712 (95 % CI: 0.603–0.821), and both 2D_DL and 2.5D_DL indicators showed AUCs of 0.710 (95 % CI: 0.600–0.819) and 0.739 (95 % CI: 0.633–0.845), respectively (Table 3, Fig. 3). The results indicate that the Combined indicator consistently outperformed individual indicators, and these findings highlight the potential utility of combining multiple indicators for improved diagnostic performance in clinical applications.

**Table 3:** C-index of different signature.

Clinical	Radiomics	2D_DL	2.5D_DL	Combined	Cohort
0.797(0.733–0.861)	0.764(0.696–0.832)	0.685(0.611–0.759)	0.739(0.669–0.809)	0.819(0.758–0.880)	train
0.799(0.703–0.896)	0.712(0.603–0.821)	0.710(0.600–0.819)	0.739(0.633–0.845)	0.804(0.708–0.900)	test

### 3.3 Grad-CAM

**Fig. 4:**
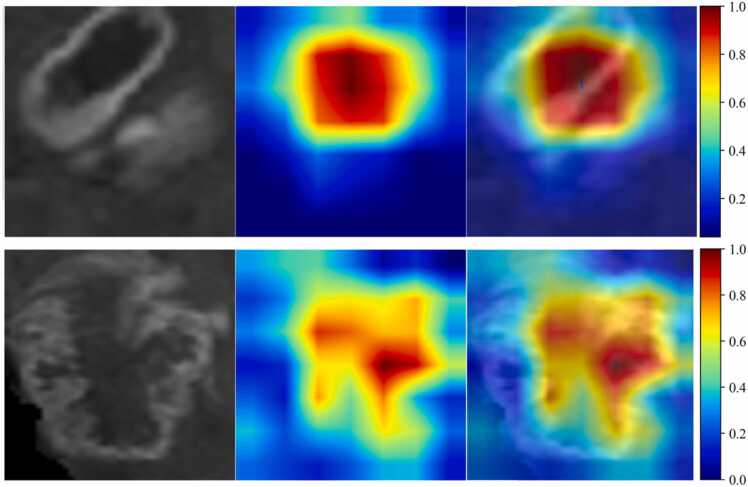
Grad-CAM visualizations for 2 representative samples.

To investigate the recognition capabilities of the deep learning models across various samples, we employed the gradient-weighted class activation mapping (Grad-CAM) technique to visualize the activations in the final convolutional layer associated with predictions of cancer-types. As shown in Fig. 4, Grad-CAM highlights the image regions that contributed most strongly to model predictions. This visualization provides clearer insights into the interpretability of the models by revealing which image features influence the models' decisions.

### 3.4 Time-dependent ROC analysis

**Fig. 5:**
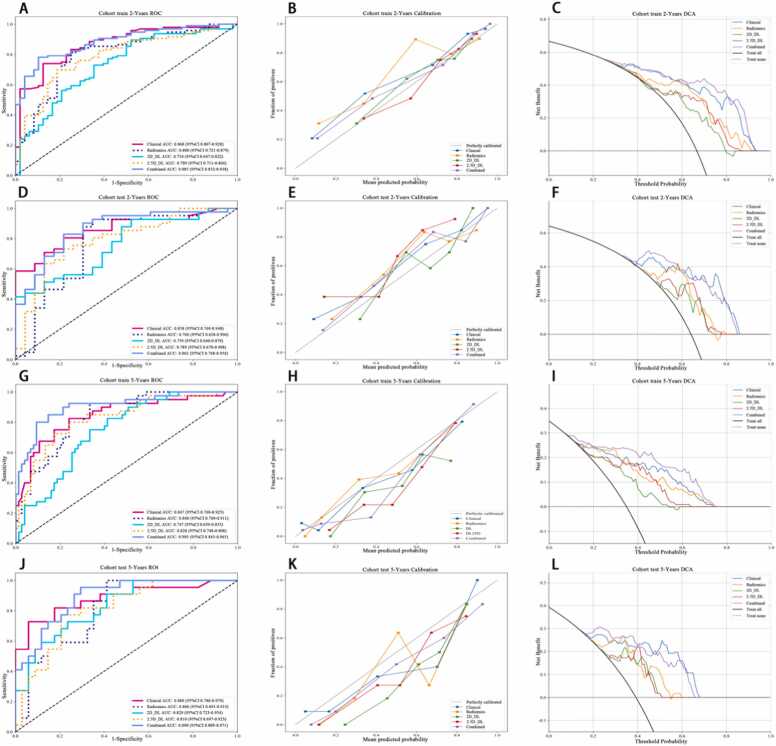
ROC Curves, Calibration Curve and DCA (decision curve analysis) in Training and Testing Cohorts for 2-Years and 5-Years Time-Dependent Analysis.

The AUC (Area Under the Curve) values for the different indicators varied across Clinical, Radiomics, 2D_DL, 2.5D_DL, and Combined models. For the Clinical model, AUC ranged from 0.850 to 0.868, indicating high discriminative performance across different survival periods (1–5 years). The Radiomics model showed AUC values between 0.759 and 0.840, suggesting moderate to high discriminative performance. The 2D_DL model exhibited lower AUC values, ranging from 0.660 to 0.765, indicating limited discriminative performance compared to other models. In contrast, the 2.5D_DL model had AUC values between 0.753 and 0.830, indicating moderate discriminative performance and representing a distinct model that integrates 2.5-dimensional input. Furthermore, the Combined models demonstrated the highest AUC values, ranging from 0.851 to 0.906, suggesting excellent discriminative performance across all survival periods. Notably, the combined models consistently outperformed individual Clinical, Radiomics, 2D_DL, and 2.5D_DL models (Table 4) and conferred greater clinical net benefits in both training and validation cohorts, as shown by decision curve analysis (DCA) (Fig. 5). The AUC analysis revealed that the combined signatures had the highest performance in predicting survival. These findings highlight the importance of combining multiple indicators that enhance predictive accuracy in survival analysis. Moreover, the consistent performance of the combined signatures across different survival periods suggests their robust predictive ability. Consequently, this consistency indicates their potential utility in clinical decision-making.

**Table 4:** Metrics in train and test cohorts for predicting the risk of survival at 1–5 Years.

models	Accuracy	AUC	95 % CI	Sensitivity	Specificity	PPV	NPV	Survival	Cohort
Clinical	0.812	0.85	0.7726–0.9274	0.832	0.733	0.925	0.524	1Years	Train
Radiomics	0.758	0.793	0.7062–0.8804	0.748	0.8	0.937	0.444	1Years	Train
2D_DL	0.799	0.66	0.5482–0.7711	0.899	0.4	0.856	0.5	1Years	Train
2.5D_DL	0.758	0.753	0.6547–0.8518	0.782	0.667	0.903	0.435	1Years	Train
Combined	0.725	0.851	0.7847–0.9167	0.681	0.9	0.964	0.415	1Years	Train
Clinical	0.785	0.861	0.7651–0.9561	0.745	0.929	0.974	0.5	1Years	Test
Radiomics	0.8	0.759	0.6149–0.9033	0.824	0.714	0.913	0.526	1Years	Test
2D_DL	0.8	0.759	0.6162–0.9020	0.843	0.643	0.896	0.529	1Years	Test
2.5D_DL	0.831	0.814	0.6740–0.9534	0.882	0.643	0.9	0.6	1Years	Test
Combined	0.877	0.874	0.7698–0.9781	0.902	0.786	0.939	0.687	1Years	Test
Clinical	0.785	0.868	0.8073–0.9282	0.74	0.875	0.922	0.627	2Years	Train
Radiomics	0.792	0.8	0.7209–0.8789	0.812	0.75	0.867	0.667	2Years	Train
2D_DL	0.757	0.734	0.6469–0.8219	0.896	0.479	0.775	0.697	2Years	Train
2.5D_DL	0.736	0.789	0.7108–0.8664	0.698	0.812	0.882	0.574	2Years	Train
Combined	0.819	0.885	0.8319–0.9381	0.781	0.896	0.937	0.672	2Years	Train
Clinical	0.797	0.858	0.7688–0.9480	0.805	0.783	0.868	0.692	2Years	Test
Radiomics	0.828	0.766	0.6276–0.9037	0.927	0.652	0.826	0.833	2Years	Test
2D_DL	0.625	0.759	0.6405–0.8781	0.415	1	1	0.489	2Years	Test
2.5D_DL	0.75	0.789	0.6699–0.9080	0.732	0.783	0.857	0.621	2Years	Test
Combined	0.812	0.862	0.7680–0.9563	0.829	0.783	0.872	0.72	2Years	Test
Clinical	0.794	0.862	0.8012–0.9228	0.785	0.806	0.838	0.746	3Years	Train
Radiomics	0.809	0.833	0.7641–0.9027	0.873	0.726	0.802	0.818	3Years	Train
2D_DL	0.723	0.765	0.6843–0.8449	0.835	0.581	0.717	0.735	3Years	Train
2.5D_DL	0.766	0.83	0.7625–0.8978	0.747	0.79	0.819	0.71	3Years	Train
Combined	0.83	0.901	0.8525–0.9486	0.835	0.823	0.857	0.797	3Years	Train
Clinical	0.812	0.875	0.7873–0.9636	0.676	0.967	0.958	0.725	3Years	Test
Radiomics	0.797	0.782	0.6619–0.9028	0.941	0.633	0.744	0.905	3Years	Test
2D_DL	0.734	0.754	0.6347–0.8731	0.941	0.5	0.681	0.882	3Years	Test
2.5D_DL	0.766	0.783	0.6697–0.8970	0.706	0.833	0.828	0.714	3Years	Test
Combined	0.859	0.885	0.8009–0.9697	0.941	0.767	0.821	0.92	3Years	Test
Clinical	0.805	0.865	0.8026–0.9275	0.839	0.775	0.765	0.846	4Years	Train
Radiomics	0.789	0.84	0.7724–0.9073	0.903	0.69	0.718	0.891	4Years	Train
2D_DL	0.692	0.751	0.6685–0.8331	0.806	0.592	0.633	0.778	4Years	Train
2.5D_DL	0.767	0.814	0.7416–0.8872	0.774	0.761	0.738	0.794	4Years	Train
Combined	0.857	0.906	0.8551–0.9568	0.79	0.915	0.891	0.833	4Years	Train
Clinical	0.857	0.898	0.8168–0.9784	0.759	0.941	0.917	0.821	4Years	Test
Radiomics	0.778	0.812	0.7053–0.9195	1	0.588	0.674	1	4Years	Test
2D_DL	0.698	0.791	0.6823–0.8999	1	0.441	0.604	1	4Years	Test
2.5D_DL	0.762	0.78	0.6655–0.8943	0.724	0.794	0.75	0.771	4Years	Test
Combined	0.825	0.899	0.8258–0.9713	0.966	0.706	0.737	0.96	4Years	Test
Clinical	0.783	0.847	0.7690–0.9254	0.825	0.76	0.647	0.891	5Years	Train
Radiomics	0.757	0.84	0.7693–0.9113	0.925	0.667	0.597	0.943	5Years	Train
2D_DL	0.696	0.747	0.6585–0.8355	0.75	0.667	0.545	0.833	5Years	Train
2.5D_DL	0.774	0.828	0.7483–0.9077	0.8	0.76	0.64	0.877	5Years	Train
Combined	0.87	0.905	0.8452–0.9648	0.8	0.907	0.821	0.895	5Years	Train
Clinical	0.857	0.88	0.7801–0.9792	0.727	0.941	0.889	0.842	5Years	Test
Radiomics	0.75	0.806	0.6931–0.9192	1	0.588	0.611	1	5Years	Test
2D_DL	0.714	0.829	0.7235–0.9343	0.909	0.588	0.588	0.909	5Years	Test
2.5D_DL	0.786	0.81	0.6973–0.9230	0.773	0.794	0.708	0.844	5Years	Test
Combined	0.804	0.89	0.8094–0.9714	0.955	0.706	0.677	0.96	5Years	Test

AUC: Area Under Curve; CI: Confidence Interval; PPV: Positive Predictive Value; NPV: Negative Predictive Value.

### 3.5 Deep learning radiomics nomogram (DLRN) construction

**Fig. 6:**
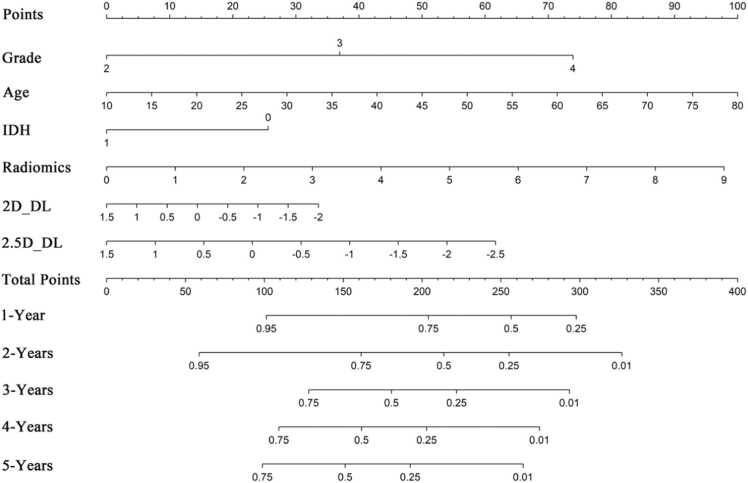
The constructed nomogram for the combined model.

The chart presents multiple indicators, including Points, Grade, Age, IDH, Radiomics, 2D_DL, 2.5D_DL, Total Points, and survival probabilities for 1- to 5- year periods. Points range from 0 to 100. Grade takes values of 2, 3, and 4; Age ranges from 10 to 80; and IDH takes values of 0 and 1. Radiomics ranges from 0 to 9; 2D_DL and 2.5D_DL take values from −2–1.5; and Total Points range from 0 to 400. The survival probabilities for 1- to 5-year periods correspond to different cutoff thresholds of Total Points (Fig. 6). The 1-Year indicator shows a relatively wide range of survival probabilities, indicating potential variability. However, the 5-Year indicator demonstrates more consistent and concentrated survival probability estimates, suggesting that the model's predictions for 5-Year survival are more stable. This stability implies that the model has a better ability to accurately predict long-term survival, making it the best-performing model among the analyzed indicators.

## 4 Discussion

This research introduces a multi-source deep learning framework aimed at predicting survival outcomes for glioma patients. The framework utilizes T1-weighted contrast-enhanced MRI (T1CE) and integrates both 2D and pseudo-3D deep learning architectures — the latter, also known as 2.5D, analyzes multiple adjacent 2D slices to capture volumetric information without full 3D processing [13]. This design leverages the strengths of each imaging dimension. Moreover, our approach shows that combining deep learning-extracted radiomic features with established clinical and molecular biomarkers significantly improves survival prediction. This outperforms conventional imaging techniques and common clinical assessment methods in neuro-oncology.

The innovative 2.5D model represents a significant technical leap, effectively capturing the three-dimensional structure of tumors while addressing the computational challenges associated with full 3D convolutional networks [14], [15], [16]. By encoding orthogonal planes as multi-channel inputs, this architecture maintains crucial spatial relationships without the need for isotropic volumetric processing. This makes it a practical solution for clinical settings with limited resources where high-resolution 3D analysis is not feasible [17]. These models successfully address challenges posed by datasets with small sample sizes, a common issue in medical imaging caused by the high cost and complexity of data acquisition. These advancements not only pave the way for more sophisticated and accurate diagnostic tools but also demonstrate how deep learning technologies are evolving to become more integrated and context-aware. Building upon the 2.5D model architecture, we employed transfer learning from pre-trained convolutional neural networks (CNNs), such as ResNet50 and DenseNet121, to extract subtle malignancy features indicative of tumor aggressiveness. This approach resulted in strong prognostic performance, outperforming unimodal benchmarks—models that rely on a single data modality.

We implemented a rigorous feature refinement process by applying stability thresholding (ICC ≥ 0.85) to reduce variability caused by segmentation, while using sparsity-constrained Cox modeling (L1-penalized regression) to eliminate redundant features without losing biological significance. This dual-filter strategy contrasts with previous radiomic studies that often led to overfitting in diverse patient cohorts due to insufficient feature selection. We improved predictive accuracy and aligned our model with current glioma classification systems by explicitly including molecular markers such as IDH mutation status and 1p/19q codeletion, along with clinical variables. This integration with established classification systems enhances its applicability in clinical practice [18], [19]. To evaluate the model, internal validation was performed using demographically and clinically balanced cohorts divided into training and testing sets at a ratio of 7:3. All baseline characteristics showed no significant differences (p-values > 0.05) between the training and testing groups, confirming the model's generalizability. Time-dependent ROC analyses further supported these results, demonstrating area under the curve (AUC) values exceeding 0.80 across 1- to 5- year survival horizons, consistently outperforming both isolated radiomic features and clinicopathological predictors. Collectively, these results suggest that multi-source integration of radiomic features, molecular, and clinical data captures the biological complexity of gliomas more effectively than single-modality feature sets.

Our findings are consistent with emerging evidence indicating that deep learning techniques demonstrate a superior predictive performance compared to traditional linear models, such as the Cox proportional hazards model. These techniques are particularly effective in identifying nonlinear determinants of survival. The superiority of the 2.5D architecture over 2D methods aligns with new multiplanar sampling paradigms, which better characterize tumor heterogeneity [20]. Importantly, this approach advances the field of survival analysis by integrating clinical and imaging data in a bidirectional manner. This means that information flows both ways between these data types, setting it apart from studies that focus solely on imaging features.

Additionally, the combined model showed better calibration performance, as indicated by the Hosmer-Lemeshow test. It also demonstrated increased clinical utility, as shown by the decision curve analysis. The improvements in calibration and clinical utility directly address the discrepancy between algorithmic performance and clinical implementation by enhancing the model's calibration accuracy. This improvement, in turn, supports practical aspects such as clinical decision-making, patient management, and treatment planning. Furthermore, this development aligns with Decuyper's emphasis on the need for prognostic tools that balance statistical rigor with clinical practicality [21].

Our study has several limitations. Firstly, the retrospective nature of data collection may introduce selection bias and limit the ability to infer causality from the observed relationships. Moreover, the generalizability of our findings to other populations or settings remains to be validated through well-designed prospective studies. Secondly, constraints related to imaging techniques, such as limited resolution or variability in feature extraction methods, could affect the reproducibility of results in different clinical environments. Thirdly, this study included only T1CE sequences and did not incorporate advanced imaging modalities such as diffusion-weighted imaging (DWI) and perfusion-weighted imaging (PWI). These advanced techniques can evaluate microenvironmental factors like cellularity and angiogenesis, and their inclusion would significantly improve the biological relevance of the findings [22]. While internal validation helps reduce selection bias, it is crucial to conduct external tests across various institutions with differing imaging protocols to confirm the robustness of the results across different scanners. Future research could incorporate treatment-response biomarkers, such as MGMT methylation status, which would help refine risk-adapted therapeutic strategies and build on the foundational work by Shreyesh, who demonstrated the prognostic value of integrating molecular markers with imaging features [23].

This framework provides a non-invasive preoperative decision-support tool that is particularly beneficial in cases where diagnoses are ambiguous or when surgery is not advisable. By generating personalized survival estimates, the framework helps to select appropriate management strategies across the risk spectrum, ranging from active surveillance for low-risk cases to aggressive adjuvant therapies for high-risk patients. To enhance the clinical feasibility of the approach, standardized preprocessing techniques such as isotropic resampling, are employed, along with open-source tools like PyRadiomics and PyTorch.

## 5 Conclusions

In conclusion, combining 2.5D deep learning with rigorous radiomic data processing significantly improves survival prediction for glioma patients, and adding multi-source data further enhances this predictive capability. This research addresses challenges related to both generalizability and clinical interpretability, which facilitates the adoption of AI-driven prognostic systems in routine neuro-oncology. Consequently, these advancements in predictive modeling enable the development of personalized therapeutic approaches, including treatment plans based on individual patient profiles.

## CRediT authorship contribution statement

**jin kai:** Writing – original draft, Software, Project administration, Methodology, Formal analysis, Conceptualization. **Caixing Sun:** Writing – review & editing, Investigation, Funding acquisition, Data curation. **Liang Xia:** Writing – review & editing, Funding acquisition.

## Ethical statement

Ethical approval was not required for this study because it utilized publicly available, de-identified data from the Chinese Glioma Genome Atlas (CGGA). These datasets contain aggregated population-level statistics and do not involve individual patient information or direct human/animal experimentation.

## Funding

The present study was supported by Zhejiang Provincial National Science Foundation of China and Zhejiang Health Science and Technology Plan (LY21H160007, LY22H160043, and 2022KY679)，and the Medical Science and Technology Project of Zhejiang Province (2022RC116, 2024KY805).

## Declaration of Competing Interest

All authors have no conflicts of interest to declare.

## Data Availability

The radiomics and clinical data used to support this study are deposited in CGGA (http://www.cgga.org.cn/).
